# Patient-reported outcomes in studies of STereotactic arrhythmia Radioablation for ventricular tachycardia (PRO-STAR) – A scoping review on behalf of the STOPSTORM.Eu consortium

**DOI:** 10.1016/j.ctro.2026.101271

**Published:** 2026-08-22

**Authors:** Alexander Fabian, Wiert F. Hoeksema, Marcin Miszczyk, Pieter G. Postema, Oliver Weiner, Marta Perin, Etienne Pruvot, Matteo Anselmino, David Krug, Oliver Blanck, Chiara Crico

**Affiliations:** a Department of Radiation Oncology, University Hospital Schleswig-Holstein, Kiel, Germany; b Department of Clinical and Experimental Cardiology, Heart Center, Amsterdam Cardiovascular Sciences, Heart Failure and Arrhythmias, Amsterdam UMC Location University of Amsterdam, Amsterdam, the Netherlands; c Comprehensive Cancer Center, Department of Urology, Medical University of Vienna, Vienna, Austria; d Collegium Medicum - Faculty of Medicine, WSB University, Dabrowa Górnicza, Poland; e University Library Kiel, Christian-Albrechts-University Kiel, Kiel, Germany; f Legal Medicine and Bioethics, Azienda USL-IRCCS di Reggio Emilia, Reggio Emilia, Italy; g Arrhythmia Unit, Cardiovascular Department, Centre Hospitalier Universitaire Vaudois and University of Lausanne, Lausanne, Switzerland; h Division of Cardiology, Cardiovascular and Thoracic Department, “Città della Salute e della Scienza” Hospital, Turin, Italy; i Department of Medical Sciences, University of Turin, Turin, Italy; j Fondazione IRCCS Istituto Nazionale Tumori, Milan, Italy

**Keywords:** Stereotactic arrhythmia radioablation, STAR, Ventricular tachycardia, Cardiac arrhythmias, Stereotactic body radiotherapy, SBRT, Patient-reported Outcomes, Quality of Life, Consortium, EU Horizon 2020

## Abstract

Although quality of life is a key outcome in cardiovascular disease, it is unclear how Patient-Reported Outcomes (PROs) are employed in trials of STereotactic Arrhythmia Radioablation (STAR). We conducted a scoping review and found that only 55% of STAR trials employ PROs and that these were almost exclusively generic measures.

## Highlights

• Protocol-based scoping review on PRO research in published and registered STAR trials.
• 55% of published and 56% of registered STAR trials assessed PROs.
• No published and 7% of registered trials used disease specific PROMs.
• No trial used STAR-specific PROMs.
• This data indicate possible gaps in PRO assessment in STAR trials.

## 1 Introduction

STereotactic Arrhythmia Radioablation (STAR) is an emerging treatment modality for patients with refractory Ventricular Tachycardia (VT). VT arises from a malfunction of cardiac electric circuits potentially originating from both, ischemic and non-ischemic heart disease [1]. VT is associated with significant mortality and impaired Health-Related Quality of Life (HRQoL) [2], [3]. Guideline-directed therapies include an implantable cardioverter-defibrillator (ICD), antiarrhythmic drugs, and catheter ablation [4]. Early STAR trials reported a reduction of VT episodes, ICD shocks, and antiarrhythmic drug dose in patients with refractory VT [5]. The STOPSTORM.eu consortium is a collaborative project pooling large volume of data on patients treated with STAR across many European centers [6], [7].

Patient-Reported Outcomes (PROs) are outcomes directly reported by the patient without interpretation by proxy [8]. PROs are commonly assessed by validated multi-item questionnaires, so called Patient-Reported Outcome Measures (PROMs). PROMs capture different aspects of patient perspectives, symptoms and/or HRQoL. Generic PROMs focus on broadly applicable aspects of HRQoL, whereas disease-specific PROMs focus on HRQoL relevant to a subset of patients such as those with cardiovascular disease or, even more specific, with arrhythmia. The Short Form Health Survey 36 (SF-36) is an example for a generic PROM that measures general aspects of HRQoL such as physical functioning, pain or mental health [9]. Cardiovascular and arrhythmia-specific PROMs have been validated as well [10]. The Arrhythmia-Specific Questionnaire in Tachycardia and Arrhythmia (ASTA), for example, targets arrythmia-specific symptoms and HRQoL issues [11]. Cardiovascular societies have promoted the use of PROs in prospective trials since 2014 to inform patient-centered care and eventually improve the quality of patient care [12]. In the context of STAR, HRQoL is a particularly relevant outcome dimension. STAR is predominantly used in patients with end-stage heart failure and refractory VT, a population characterized by severe symptom burden and reduced life expectancy. As STAR is a palliative treatment, measuring HRQoL may deliver a more meaningful and patient-centered approach to assess the value of STAR as compared to efficacy-related endpoints focusing on the reduction of VT burden.

To our knowledge, the implementation of PROs in STAR trials has not yet been studied systematically. Therefore, we performed a scoping review on research practice of PROs in prospective STAR trials.

## 2 Materials and methods

We conducted a scoping review on research practices of PROs within STAR trials following the JBI methodology for scoping reviews [13]. The JBI methodology for scoping reviews comprises a structured approach that uses predefined research questions and search criteria to map the evidence or research practice of a specific area based on a systematic literature search and transparent reporting. Our protocol was pre-registered on the Open Science Framework platform (https://doi.org/10.17605/OSF.IO/28HDJ, registration date: 13/Aug/2025) (Supplementary Material Protocol). The primary objective was to investigate the prevalence of PRO use in STAR trials. Secondary objectives aimed to explore methodological aspects of PROs and PROMs. We extensively searched Pubmed/MEDLINE, Embase, Web of Science, and Cochrane Central Register of Controlled Trials for full text published trials and trial protocols from inception to 3/Sep/2025 without restrictions in language. The registry”ClinicalTrials.gov” was searched for registered trials of any status without results published in full text (Supplementary Material Protocol). The search strategy was adapted from a previously published study and the strategy is shown in the supplementary material (Supplementary Material Protocol) [14]. The web-application Rayyan was used for reference management (Rayyan Systems Inc. [2026], Cambridge, USA). Prospective trials as per National Institutes of Health (NIH) definition (i.e., “a research study in which one or more human subjects are prospectively assigned to one or more interventions (...) to evaluate the effects of those interventions on health-related biomedical or behavioral outcomes”) were eligible. Eligibility of prospective trials based on the NIH definition was judged by each collaborator involved in literature screening taking into account all aspects of the definition (i.e., prospective assignment to intervention & human subject & health-related biomedical/behavioral outcome). Hand search of all reviews identified during reference screening did not yield additional eligible trials. At least two collaborators conducted each step independently, including title/abstract screening, full text screening and data extraction via predefined data extraction forms. Conflicts were resolved through discussion. Key extracted variables included study characteristics (e.g. randomization), sample characteristics (e.g. sample size), and information on PROs (e.g. use, names and types of PROMs, primary vs. secondary endpoint). We used descriptive statistics to display trial characteristics and logistic regression to investigate trends over time in years and the use of PROs by trials. Analyses were performed using JASP v0.19.3 (JASP Team [2025], Amsterdam, The Netherlands).

## 3 Results

Of 2357 identified references, 1427 references were screened for abstracts after removal of duplicates as shown in the PRISMA flow chart (Supplementary Fig. 1). Of 69 published trials and 30 registered trials screened for full texts, 11 published trials and 27 trial registrations were included in the analysis. References of elected trials are shown in Supplementary Document 1. Three full text protocols were available for registered trials.

Characteristics of published trials are shown in Supplementary Table 1. No trial was randomized, 18% (*n* = 2) were multicenter studies, and the median patient sample size was 6 (interquartile range (IQR): 6). Of 88 patients in total, the median patient age was 67 years (IQR: 5), 9% (*n* = 8) were female patients, and the New York Heart Association class was II in 28% (*n* = 25) and III in 30% (*n* = 26) of the patients, respectively. The characteristics of registered trials are shown in Supplementary Table 2. Of these, 30% (*n* = 8) were randomized and 15% (*n* = 4) were multicenter studies, respectively. The median planned sample size was 21 (IQR: 28).

**Fig. 1:**
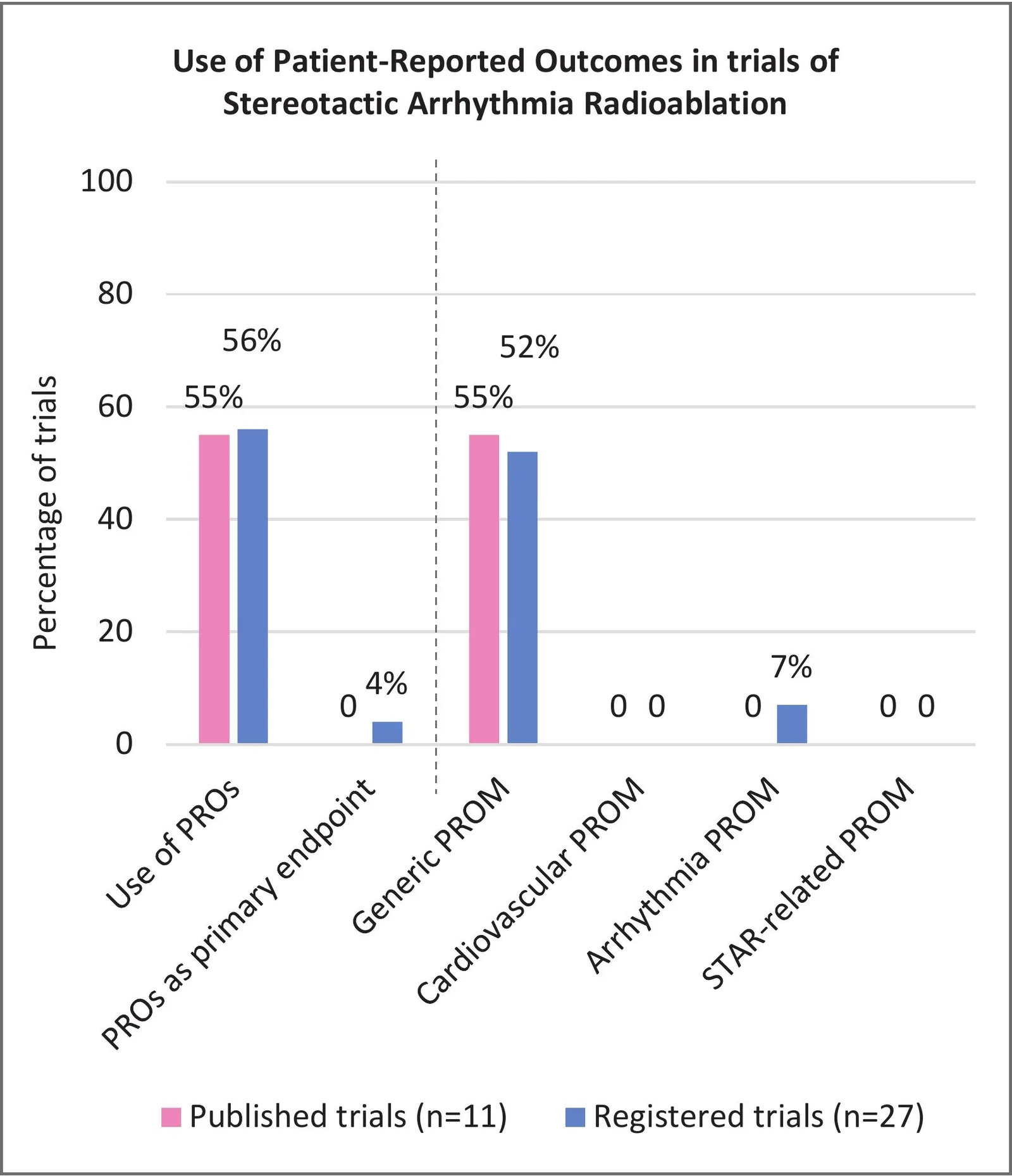
Use of Patient-Reported Outcomes (PROs) in trials of Stereotactic Arrhythmia Radioablation for ventricular tachycardia. The histogram shows relative frequencies for published trials (*n* = 11) and registered trials (*n* = 27), respectively. One registered trial employed PROs but did not report the measure used. Abbreviations: PROs, Patient-Reported Outcomes; PROM, Patient-Reported Outcome Measure.

Concerning our primary objective on the prevalence of PRO use in STAR trials, 55% (*n* = 6) of the published trials and 56% (*n* = 15) of the registered trials used PROs, respectively (Fig. 1). There was neither a trend over time in years for the use of PROs in published trials (odds ratio [OR], 0.84 [95% CI, 0.39–1.81]; *p* = 0.66) nor in registered trials (OR, 0.76 [95% CI, 0.49–1.18]; *p* = 0.22) as per logistic regression.

**Fig. 2:**
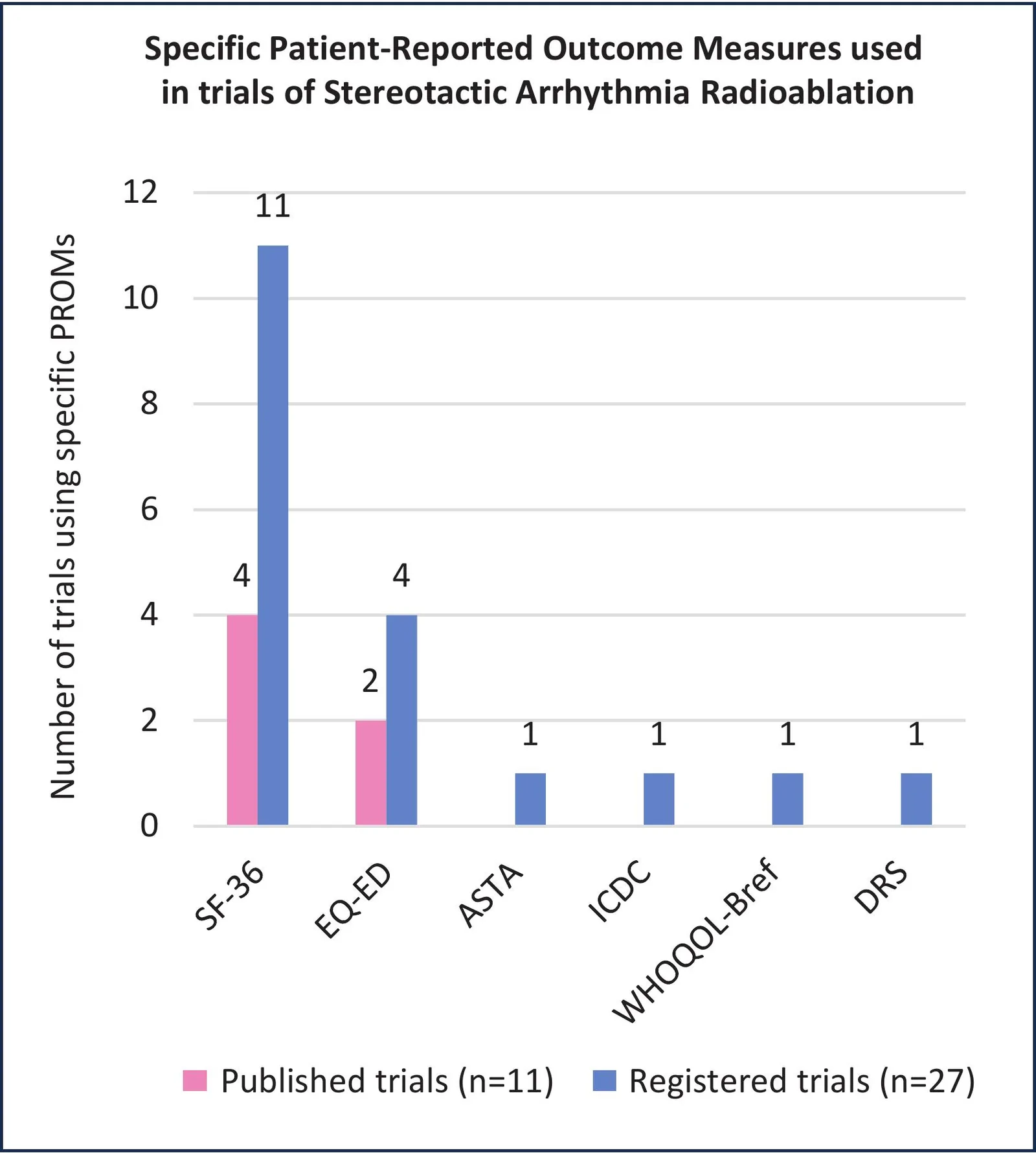
Specific Patient-Reported Outcomes Measures (PROMs) used in trials of Stereotactic Arrhythmia Radioablation for ventricular tachycardia. The histogram shows absolute frequencies for each PROM and for published (*n* = 11) and registered trials (*n* = 27), respectively. One registered trial employes Patient-reported Outcomes but did not report the PROM used. Abbreviations: ASTA, Arrhythmia-Specific questionnaire in Tachycardia and Arrhythmia; DRS, Decision Regret Scale; EQ-5D, EuroQol 5 Dimensions; ICDC, Implantable Cardioverter-Defibrillator Concerns; SF-36, Short Form-36; WHOQOL-Bref, World Health Organization Quality of Life.

Concerning our secondary objective on methodological aspects of PROs in STAR trials, no published trial used PROs as primary endpoint (Fig. 1). One registered trial used PROs as co-primary endpoint (NCT05696522). Generic PROMs were used by 55% (*n* = 6) of the published trials and 52% (*n* = 14) of the registered trials. Overarching cardiovascular PROMs were not used. No published trial and 7% (*n* = 2) of the registered trials used arrhythmia-specific PROMs. No trial used a STAR-related PROM. The PROMs used are shown in Fig. 2. The exact PROM used was unknown concerning one registered trial that reported to assess PRO. Among those trials who used PROs, one PROM per trial was used concerning all published trials. Concerning registered trials, 67% (*n* = 10) used one, 20% (*n* = 3) used two, and 7% (*n* = 1) used four PROMs per trial, respectively. All trials using PROs assessed them longitudinally, used validated PROMs (except for one registered trial with unknown PROM), and analyzed PROs on an exploratory basis.

## 4 Discussion

In this scoping review on PRO use in STAR trials, we have found that only one out of two trials investigates PROs. Trials almost exclusively used generic PROMs in lieu of cardiovascular or arrhythmia-specific PROMs. Only one registered trial used PROs as a (co-)primary endpoint.

The rate of PRO adoption in STAR trials appears comparable to other contexts such as oncological palliative radiotherapy trials [15], [16]. Compared to trials of catheter ablation for VT, the adoption of PROs in STAR trials appears even superior as a recent systematic review identified only one clinical trial of catheter ablation reporting PROs [17]. However, our data demonstrates that the goal of comprehensive PRO assessment in STAR trial of VT is not yet met [12]. The incomplete adoption of PROs raises important questions about how we define and measure treatment efficacy in STAR. With only half of trials including PROs, there is a tangible risk of having an incomplete understanding of STAR benefits if patients' lived experience continues to be overlooked. Clinical outcomes such as VT burden reduction may provide an incomplete picture of efficacy; true therapeutic benefit must integrate both clinical endpoints and PROs. Can we genuinely claim ‘benefit’ without understanding how patients experience the reduced VT burden? The predominant use of generic PROMs (e.g. SF-36, EQ-5D) adds to this concern, as these instruments fail to capture VT- and ICD-specific concerns such as shock anxiety, the psychological impact of living with an ICD, or the loss of sense of control that may characterize this patient population.

Apparently, there are barriers to the adoption of comprehensive PRO assessment in STAR trials. Potential barriers could speculatively be a limited awareness for the importance of PROs or limitations in funding as most trials had academic sponsorship. Another reason for the incomplete use of PROs could have been the fact that most trials were early phase non-randomized studies, though the value of PROs has also been emphasized in early phase oncological studies [18].

Beyond methodological considerations, the underutilization of PROs in STAR trials may have ethical implications [7]. PROs represent a concrete operationalization of fundamental ethical principles. From a beneficence perspective, PROs reveal which dimensions of suffering are actually modified by STAR, providing essential evidence to determine whether the intervention truly benefits patients. Regarding autonomy, informed decision-making requires realistic expectations about quality of life—patients need to know not only whether they will have fewer device-detected events, but whether they might feel better. This is particularly critical given the double vulnerability faced by VT patients: physical fragility due to electrical cardiac instability and psychosocial fragility. PROs can help make this vulnerability visible and actionable.

Opportunities to further leverage the role of STAR for VT could be the design of studies with PROs as primary endpoint to investigate the impact of STAR on HRQoL. Further, the validation of arrhythmia-specific PROMs in patients treated with STAR or even the development of STAR-related PROMs would be useful given that STAR differs from other arrhythmia treatments such as in terms of potential side effects including radiation-induced esophagitis or pneumonitis. It is a limitation of our protocol-based scoping review that we only found few published full text protocols of registered trials. This may have limited the depth of data capture, as we had to rely on ClinicalTrials.gov registry entries, where reporting may be incomplete. Another limitation is the overall small number of trials eligible for our review. Finally and in line with the methodology of scoping reviews, we did not conduct formal critical appraisal of included trials. Hence, our findings should only be interpreted as a description of current research practice.

In conclusion, as only one out of two STAR trials adopted PROs, future studies should promote the use of PROs and incorporate disease-specific PROMs in their study design. Comprehensive PRO assessment should be considered a standard component of trial design rather than an optional addition.

## Availability of data and material

The data that support the findings of this study are available from the corresponding author upon reasonable request.

## CRediT authorship contribution statement

**Alexander Fabian:** Writing – review & editing, Writing – original draft, Project administration, Methodology, Investigation, Formal analysis, Data curation, Conceptualization. **Wiert F. Hoeksema:** Writing – review & editing, Writing – original draft, Investigation. **Marcin Miszczyk:** Writing – review & editing, Methodology. **Pieter G. Postema:** Writing – review & editing. **Oliver Weiner:** Writing – review & editing, Methodology. **Marta Perin:** Writing – review & editing, Investigation. **Etienne Pruvot:** Writing – review & editing. **Matteo Anselmino:** Writing – review & editing. **David Krug:** Writing – review & editing. **Oliver Blanck:** Writing – review & editing, Funding acquisition. **Chiara Crico:** Writing – review & editing, Writing – original draft, Supervision, Investigation, Conceptualization.

## Ethics approval

Not applicable given the study design of a scoping review.

## Funding

The study has received funding from the European Union's Horizon 2020 research and innovation program under grant agreement No 945119 (STOPSTORM.eu Consortium).

## Declaration of competing interest

The authors declare the following financial interests/personal relationships which may be considered as potential competing interests: AF received honoraria from Merck Sharp and Dohme as well as a research award from Lilly Germany Foundation. DK has received honoraria from Astra Zeneca, best practice onkologie, ESO, ESMO, Gilead, med update, Merck Sharp & Dohme, Novartis, onkowissen, and Pfizer, served on advisory boards for Gilead and has received institutional research funding from Stiftung Deutsche Krebshilfe and Merck KGaA. P.G.P. has received funding from the Dutch Heart Foundation (grant no. 03-003-2021-T061). MA is consultant for Johnson & Johnson and Boston Scientific.

## Appendix A Supplementary data

Supplementary material 4 Supplementary Document 1

Supplementary material 5 Protocol
