# Supplementary material for: Patient-reported outcomes in studies of STereotactic arrhythmia Radioablation for ventricular tachycardia (PRO-STAR) – A scoping review on behalf of the STOPSTORM.Eu consortium
Source: Clin Transl Radiat Oncol. 2026 Aug 22;61:101271. doi: 10.1016/j.ctro.2026.101271 (PMC13544081; doi:10.1016/j.ctro.2026.101271)
Supplement: Supplementary file 1 [file mmc1.pdf]

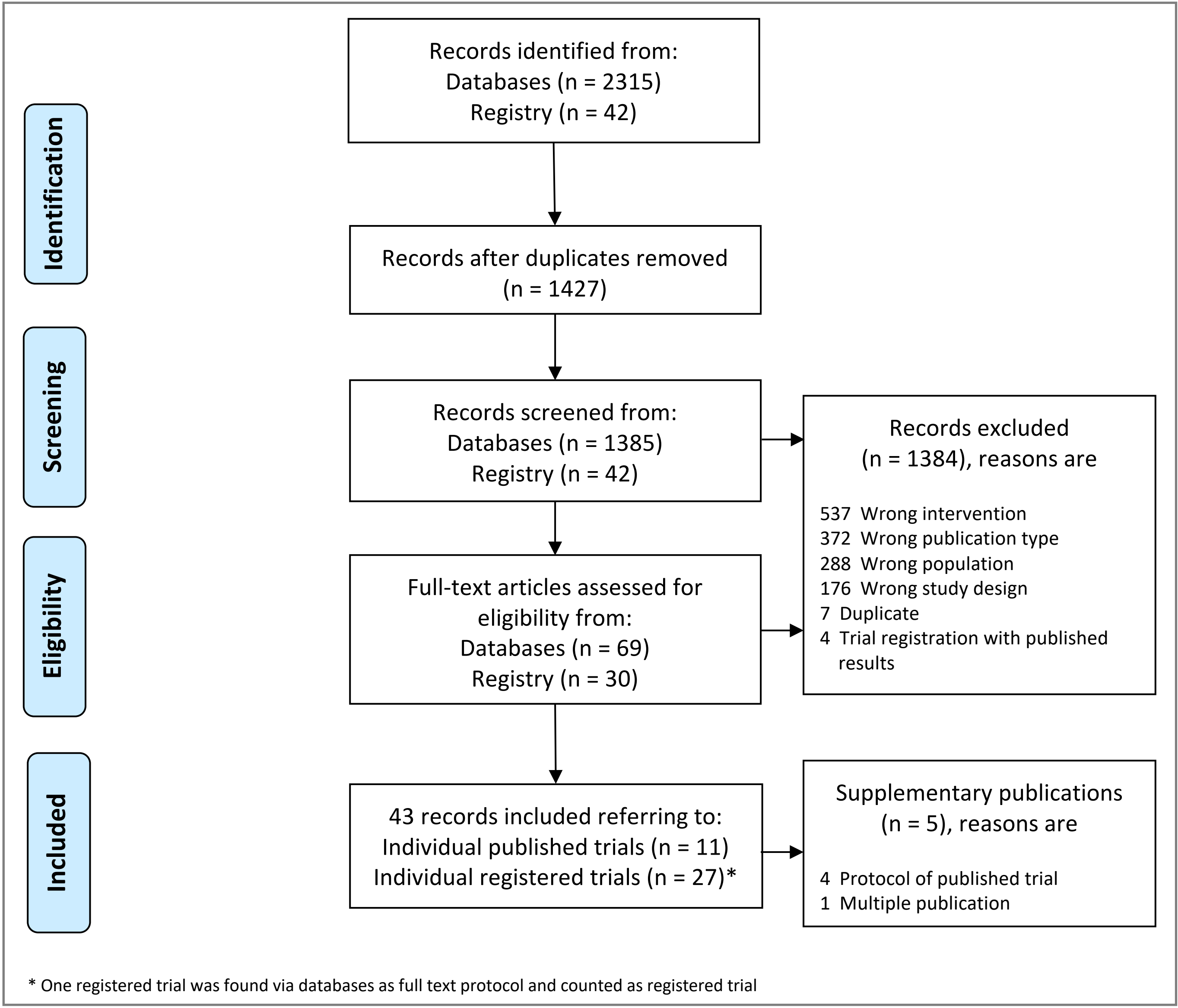

Supplementary Figure 1 PRISMA Flow Chart
