# Supplementary material for: Patient-reported outcomes in studies of STereotactic arrhythmia Radioablation for ventricular tachycardia (PRO-STAR) – A scoping review on behalf of the STOPSTORM.Eu consortium
Source: Clin Transl Radiat Oncol. 2026 Aug 22;61:101271. doi: 10.1016/j.ctro.2026.101271 (PMC13544081; doi:10.1016/j.ctro.2026.101271)
Supplement: Supplementary file 2 [file mmc2.pdf]

Supplementary Table 1 Study and patient characteristics of 11 included published trials of Stereotactic Arrhythmia Radioablation (STAR) for patients with ventricular tachycardia. Absolute numbers are given in brackets. Abbreviations: IQR, interquartile range; NYHA, New York Heart Association

**Study characteristics**		Studies 100% (11)
Location	Europe	55% (6)
	Asia	27% (3)
	Northern America	18% (2)
Randomized trial	Yes	0
	No	100% (11)
Multicenter trial	Yes	18% (2)
	No	82% (9)
Patient sample size		Median: 6; IQR: 6
Stage of publication	Interim	45% (5)
	Final	55% (6)
Sponsor	Academic	27% (3)
	Industry	9% (1)
	Unknown	64% (7)
Treatment platform	C-Arm Linac	82% (9)
	CyberKnife	9% (1)
	MR-Linac	9% (1)
**Patient characteristics**		Patients 100% (88)
Age		Median: 67; IQR: 5
Sex	Female	9% (8)
	Male	91% (80)
NYHA Class	I	3% (3)
	II	28% (25)
	III	30% (26)
	IV	7% (7)
	Missing	31% (27)
Cardiac disease	Ischemic	59% (52)
	Non-ischemic	41% (36)
