# Supplementary material for: Patient-reported outcomes in studies of STereotactic arrhythmia Radioablation for ventricular tachycardia (PRO-STAR) – A scoping review on behalf of the STOPSTORM.Eu consortium
Source: Clin Transl Radiat Oncol. 2026 Aug 22;61:101271. doi: 10.1016/j.ctro.2026.101271 (PMC13544081; doi:10.1016/j.ctro.2026.101271)
Supplement: Supplementary file 3 [file mmc3.pdf]

Supplementary Table 2 Characteristics of 27 included unpublished registered trials of Stereotactic Arrhythmia Radioablation (STAR) for patients with ventricular tachycardia. Absolute numbers are given in brackets. Abbreviations: IQR, interquartile range

**Study characteristics**		Studies 100% (27)
Location	Europe	48% (13)
	Northern America	44% (12)
	Asia	4% (1)
	Oceania	4% (1)
Randomized trial	Yes	30% (8)
	No	70% (19)
Multicenter trial	Yes	15% (4)
	No	85% (23)
Planned patient sample size		Median: 21; IQR: 28
Sponsor	Academic	93% (25)
	Industry	4% (1)
	Unknown	4% (1)
Trial status	Ongoing	56% (15)
	Closed	15% (4)
	Not yet recruiting	11% (3)
	Withdrawn	11% (3)
	Unknown	7% (2)
Full protocol available	Yes	11% (3)
	No	89% (24)
Actual study start date	2017-2019	11% (3)
	2020-2022	30% (8)
	2023-2025	59% (16)
