# Supplementary material for: Patient-reported outcomes in studies of STereotactic arrhythmia Radioablation for ventricular tachycardia (PRO-STAR) – A scoping review on behalf of the STOPSTORM.Eu consortium
Source: Clin Transl Radiat Oncol. 2026 Aug 22;61:101271. doi: 10.1016/j.ctro.2026.101271 (PMC13544081; doi:10.1016/j.ctro.2026.101271)
Supplement: Supplementary file 4 [file mmc4.pdf]

Supplementary Document 1

Included registered trials

Trial registration number
jRCTs042230030*
NCT03349892
NCT03601832
NCT04065802
NCT04162171
NCT04334811
NCT04392193
NCT04612140
NCT04757688
NCT05047198
NCT05084391
NCT05258422
NCT05439031
NCT05594368
NCT05696522
NCT05765175
NCT05913375
NCT05973578
NCT06294782
NCT06360939
NCT06411392
NCT06593418
NCT06743789
NCT06743789
NCT06769451
NCT06922214
NCT07017855

* this trial has been found as full text protocol via database search without published results

Included published trials

[1] Borzov E, Efraim R, Suleiman M, Bar-Deroma R, Billan S, Xie J, et al. Implementing stereotactic arrhythmia radioablation with STOPSTORM.eu consortium support: intermediate results of a prospective Israeli single-institutional trial. Strahlentherapie Und Onkologie : Organ Der Deutschen Rontgengesellschaft . [et Al] 2025;201:126–34.

[2] Ho L-T, Chen JL-Y, Hsu J-C, Chan H-M, Huang Y-C, Su M-Y, et al. Long-term results of stereotactic arrhythmia radioablation for refractory ventricular Arrhythmias-A Taiwanese population study. Journal of the Formosan Medical Association = Taiwan Yi Zhi 2024.

[3] Bianchi S, Marchesano D, Magnocavallo M, Polselli M, di Renzi P, Grimaldi G, et al. Magnetic Resonance-Guided Stereotactic Radioablation for Septal Ventricular Tachycardias. JACC Clinical Electrophysiology 2024;10:2569–80.

[4] van der Ree MH, Dieleman EMT, Visser J, Planken RN, Boekholdt SM, de Bruin-Bon RHA, et al. Non-invasive stereotactic arrhythmia radiotherapy for ventricular tachycardia: results of the prospective STARNL-1 trial. Europace : European Pacing, Arrhythmias, and Cardiac Electrophysiology : Journal of the Working Groups on Cardiac Pacing, Arrhythmias, and Cardiac Cellular Electrophysiology of the European Society of Cardiology 2023;25:1015–24.

[5] Miszczyk M, Sajdok M, Bednarek J, Latusek T, Wojakowski W, Tomasik B, et al. Stereotactic management of arrhythmia - radiosurgery in treatment of ventricular tachycardia (SMART-VT). Results of a prospective safety trial. Radiotherapy and Oncology : Journal of the European Society for Therapeutic Radiology and Oncology 2023;188:109857.

[6] Krug D, Zaman A, Eidinger L, Grehn M, Boda-Heggemann J, Rudic B, et al. Radiosurgery for ventricular tachycardia (RAVENTA): interim analysis of a multicenter multiplatform feasibility trial. Strahlentherapie Und Onkologie : Organ Der Deutschen Rontgengesellschaft . [et Al] 2023;199:621–30.

[7] Amino M, Kabuki S, Kunieda E, Hashimoto J, Sugawara A, Sakai T, et al. Interim Report of a Japanese Phase II Trial for Cardiac Stereotactic Body Radiotherapy in Refractory Ventricular Tachycardia - Focus on Target Determination. Circulation Reports 2023;5:69–79.

[8] Molon G, Giaj-Levra N, Costa A, Bonapace S, Cuccia F, Marinelli A, et al. Stereotactic ablative radiotherapy in patients with refractory ventricular tachyarrhythmia. European Heart Journal Supplements : Journal of the European Society of Cardiology 2022;24:C248–53.

[9] Carbucicchio C, Andreini D, Piperno G, Catto V, Conte E, Cattani F, et al. Stereotactic radioablation for the treatment of ventricular tachycardia: preliminary data and insights from the STRA-MI-VT phase Ib/II study. Journal of Interventional Cardiac Electrophysiology : An International Journal of Arrhythmias and Pacing 2021;62:427–39.

[10] Gianni C, Rivera D, Burkhardt JD, Pollard B, Gardner E, Maguire P, et al. Stereotactic arrhythmia radioablation for refractory scar-related ventricular tachycardia. Heart Rhythm 2020;17:1241–8.

[11] Robinson CG, Samson PP, Moore KMS, Hugo GD, Knutson N, Mutic S, et al. Phase I/II Trial of Electrophysiology-Guided Noninvasive Cardiac Radioablation for Ventricular Tachycardia. Circulation 2019;139:313–21.
