# Supplementary material for: Patient-reported outcomes in studies of STereotactic arrhythmia Radioablation for ventricular tachycardia (PRO-STAR) – A scoping review on behalf of the STOPSTORM.Eu consortium
Source: Clin Transl Radiat Oncol. 2026 Aug 22;61:101271. doi: 10.1016/j.ctro.2026.101271 (PMC13544081; doi:10.1016/j.ctro.2026.101271)
Supplement: Supplementary file 5 [file mmc5.pdf]

Patient-Reported Outcomes in studies of STereotactic Arrhythmia Radioablation (PRO-STAR) for Ventricular Tachycardia – A scoping review protocol *on behalf of the STOPSTORM.eu consortium*

Alexander Fabian[1,#], Marcin Miszczyk[2,3], Wiert F. Hoeksema[4], Pieter G. Postema[4], Oliver Weiner[5], Marta Perin[6], Etienne Pruvot[7], Matteo Anselmino[8], David Krug[1, 9], Oliver Blanck[1], Chiara Crico [6,10]

[1] Department of Radiation Oncology, University Hospital Schleswig-Holstein, Kiel, Germany
[2] Comprehensive Cancer Center, Medical University of Vienna, Vienna, Austria
[3] Collegium Medicum - Faculty of Medicine, WSB University, Dabrowa Górnicza, Poland
[4] Department of Clinical and Experimental Cardiology, Heart Center, Amsterdam UMC Location University of Amsterdam, Amsterdam
[5] University Library Kiel, Christian-Albrechts-University Kiel, Kiel, Germany
[6] Legal Medicine and Bioethics, Azienda USL-IRCCS di Reggio Emilia, Reggio Emilia, Italy
[7] Arrhythmia Unit, Cardiovascular Department, Centre Hospitalier Universitaire Vaudois and University of Lausanne, Lausanne, Switzerland
[8] Division of Cardiology, Department of Medical Sciences, Città della Salute e della Scienza di Torino Hospital, University of Turin, Turin, Italy
[9] Department of Radiotherapy and Radiation Oncology, University Hospital Hamburg-Eppendorf, Hamburg, Germany
[10] Fondazione IRCCS Istituto Nazionale Tumori, Milan, Italy

[#] Corresponding author: Alexander Fabian, MD MBA (alexander.fabian(at)uksh.de)
Department of Radiation Oncology, University Hospital Schleswig-Holstein, 24105 Kiel, Germany
Arnold-Heller-Str.3, 24105 Kiel, Tel. +49-431-500-26501; Fax +49-431-500-26548

Abstract

Objective: We conduct a scoping review to investigate current Patient-Reported Outcome (PRO) research practices in trials of Stereotactic Arrhythmia Radioablation (STAR) for Ventricular Tachycardia (VT) aiming to gain a better understanding of potential gaps and inconsistencies concerning the use of PRO.

Introduction: The heart rhythm disorder VT is associated with substantial morbidity and mortality. Standard management, including antiarrhythmic drugs or catheter ablation, may prove insufficient for some patients. STAR is a novel treatment paradigm using ionizing radiation to reduce the burden of VT and first prospective trials have reported feasibility and surrogate endpoints. To date it is unknown on a larger scale if and how PRO are employed in trials of STAR for VT despite the clinical relevance of PRO, e.g. to quantify the impact of STAR on patient's health-related quality of life and/or symptoms burden.

Inclusion criteria: We will consider literature on published and ongoing clinical trials of STAR for VT. There will be no restrictions on publication period or language.

Methods: We will follow the JBI methodology for scoping reviews. Databases to be searched will include MEDLINE, Embase, Web of Science, Cochrane Central Register of Controlled Trials, Google Scholar, and ClinicalTrials.gov. Two independent coauthors will perform abstract and full-text screening. Predefined and pilot-tested data extraction forms will be used. We will present results on research practice of PRO in graphical, tabular, and narrative form as applicable.

Keywords: Cardiovascular Disease; Cardiac Arrhythmia; SBRT; Patient-reported Outcomes; Scoping Review

Review registration
Open Science Framework (DOI: 10.17605/OSF.IO/KEQSD)

Introduction

Cardiovascular disease is a leading cause of mortality and morbidity worldwide.[1] Among various cardiovascular disease types ventricular arrhythmias are often life threatening and difficult to manage.[2] Ventricular arrhythmias are characterized by an unphysiological electrical flow causing abnormal ventricular rhythms which may lead to severe symptoms or cardiac death. Ventricular arrhythmias include premature ventricular complexes, ventricular tachycardia, and ventricular fibrillation. Clinical management of ventricular tachycardia is not limited to but includes device therapy, antiarrhythmic drugs, and catheter ablation of arrhythmogenic scars.[2] STereotactic Arrhythmia Radioablation (STAR) is a relatively new non-invasive approach for refractory ventricular tachycardia directing external beam radiation to the myocardium and/or a myocardial scar.[3] Several case series and prospective studies of STAR have been reported. These studies mainly focus on surrogate endpoints such as ventricular tachycardia burden or use of antiarrhythmic drugs although it is widely acknowledged that patients emphasize a treatment's impact on symptom burden and Health-Related Quality of Life (HRQoL).[4,5].

Patient-reported outcomes (PRO) are outcomes directly reported by the patient without interpretation by proxy.[6] PRO are commonly assessed by validated multi-item questionnaires, so called patient-reported outcome measures (PROM). Different aspects of patient perspectives, symptoms and/or HRQoL may be captured by different PROM. Generic PROM focus on broadly applicable aspects of HRQoL, whereas disease specific PROM focus on HRQoL relevant to a subset of patients such as patients with cardiovascular disease.

The important role of HRQoL and PRO has already been acknowledged in cardiology.[4] In 2014, for example, Anker and colleagues published a call to implement PRO in cardiovascular trials in order to better capture the treatment's impact on HRQoL.[7] While most cardiovascular trials have applied PRO in the context of heart failure, PROM for cardiac arrhythmias have also been developed, mainly for atrial fibrillation.[8–11]

A recent review summarizes main outcomes of STAR, but provides little to no information on the use of PRO within studies of STAR.[5] Hence, we lack a clear overview on how PRO are employed in prospective studies of STAR for ventricular tachycardia.

Therefore, we conduct a scoping review to investigate current PRO research practice in trials of STAR for ventricular tachycardia. The objective of this scoping review is to describe the research landscape concerning the use of PRO in published and ongoing prospective studies of STAR for ventricular tachycardia in terms of how commonly PRO are used, which PROM are used, and how they are employed. We thereby aim to gain a better understanding of potential gaps and inconsistencies concerning the use of PRO in studies of STAR to provide comprehensive insights into potential areas of improvement for future research paths. The rationale for conducting a scoping review on a topic that, to our knowledge, has not yet been addressed in this setting by a review is i.) our aim of broadly depicting current research practices rather than efficacy outcomes ii.) the relatively new field of research in STAR.

Review question

Specific research questions for this scoping review are:

1.) What is the prevalence and extent of PRO use in published and ongoing trials of STAR for ventricular tachycardia?

Following research questions apply for studies that include PRO and focus on how PRO are employed:
2) Which specific PROM are employed and what is their scope (e.g., generic, disease-specific for cardiovascular conditions/arrhythmias, or intervention-specific)?
3) What are the methodological approaches to PROM administration and assessment, including their mode of capture (e.g., online vs. paper-and-pencil), frequency of assessment, and reported validation status?
4) What is the role and statistical intent of PRO as study endpoints (e.g., primary vs. secondary, exploratory vs. confirmatory)?

Inclusion criteria

We will include trials with ventricular tachycardia patients (=participants) treated with any type of STAR (=context). Only prospective trials will be respected as typically only these studies are able to collect PRO (=concept) in a standardized manner. Any type of PRO will be respected, including the use of validated or unvalidated PROM. We will also include ongoing unpublished trials of STAR for ventricular tachycardia. There will be no restriction on languages as long as translations, other than English, can be achieved using ChatGPT-4 or Google Translate. Such a translation needs to be verified by a native speaker.

Types of sources

This scoping review will consider only prospective trials as per definition of the National Institutes of Health (i.e., “a research study in which one or more human subjects are prospectively assigned to one or more interventions (...) to evaluate the effects of those interventions on health-related biomedical or behavioral outcomes”).[12] Pertinent reviews that will be found during literature screening will be analysed for primary sources of eligible trials that have not been detected by our search strategy. We will consider published trials as well as registered trials. Published trials that are reported in abstract form only and have no corresponding registration or protocol available will not be considered.

Methods

The proposed scoping review will be conducted in accordance with the JBI methodology for scoping reviews. [13]

Search strategy

A predefined search strategy will be employed in order to detect references relevant to the objectives and inclusion criteria of this review. Overarchingly, the search strategy is designed to detect references on ventricular tachycardia and STAR. The search strategy will be adapted for each included database.

Databases will be searched from inception. The reference list of included sources of evidence will be screened for additional studies.

We will search Pubmed/MEDLINE, Embase, Web of Science, and Cochrane Central Register of Controlled Trials for relevant published trials. The website "clinicaltrials.gov" will be screened for registered trials that are ongoing or have not yet been reported in full text. If necessary, respective available protocols of these registered trials will be hand searched. Trial protocols of ongoing trials that are detected via Pubmed/MEDLINE, Embase, Web of Science, and Cochrane Central Register of Controlled Trials will also be respected. We will not consider sources of grey literature as we estimate that these will not be relevant to our review's objectives. The search strategy has been adapted to our review objectives from a systematic review by Miszczyk and colleagues.[5]

Study selection

Following the search, all identified citations will be collated and uploaded into a citation management software and duplicates will be removed. Titles and abstracts will be screened by two or more independent reviewers for assessment against the inclusion criteria for the review after a lead-in pilot test of relevant studies. Potentially relevant sources will be retrieved in full text. The full text of selected citations will be assessed in detail against the inclusion criteria by two or more independent reviewers. Reasons for exclusion of sources of evidence at full text stage that do not meet the inclusion criteria will be recorded and reported in the scoping review. Any disagreements that arise between the reviewers at each stage of the selection process will be resolved through discussion, or with additional reviewers. The results of the search and the study inclusion process will be reported in full in the final scoping review and presented in a PRISMA flow diagram.[14]

Data extraction

Data will be extracted from papers and registered trials included in the scoping review into predefined data extraction forms after pilot testing. Data extraction will be performed by at least two independent reviewers using MS Excel or Google Sheets. Alternatively and depending on the amount of literature, after a lead in phase of at least 15% of all full texts has shown good concordance of two independent reviewers, the remaining data extraction may be performed by a single reviewer. The data extracted will include specific details about the participants, concept, context, study methods and key findings relevant to the review questions. A PROM used in a given study will be considered "validated" in line with the definition of Churruca and colleagues, i.e. "*there was published statistical analyses establishing reliability (…) and validity of the scale(s) (…)*".[15]

Draft extraction forms are provided (see Appendix II+III). The draft data extraction forms will be modified and revised as necessary during the process of extracting data from each included evidence source. Modifications will be detailed in the scoping review. Any disagreements that arise between the reviewers will be resolved through discussion, or with additional reviewers. If appropriate, authors of papers will be contacted to request missing or additional data, where required.

Data analysis and presentation

Data will be analysed descriptively and on an exploratory basis. Comparisons of published trials and ongoing registered trials will be performed concerning the use of PRO as feasible. We will also highlight

ratios of trials using PRO versus trials not using PRO. Furthermore, the most commonly used PROM will be listed. Data will be presented in graphical, tabular and narrative form as applicable.

Funding

This project has received funding from the European Union's Horizon 2020 research and innovation programme under grant agreement no. 945119. The grant agreement supports many European stereotactic arrhythmia radioablation trials, some of which have already been published and included in this review. The funding source had no influence on the development of this protocol.

Author contributions

AF and CC conceptualized the review topic and drafted the protocol. MM and OW designed the literature search strategies. All coauthors critically appraised and edited the review protocol.

Conflicts of interest

AF received honoraria from Merck Sharp and Dohme as well as a research award from Lilly Germany Foundation. DK has received honoraria from Astra Zeneca, best practice onkologie, ESO, ESMO, Gilead, med update, Merck Sharp & Dohme, Novartis, onkowissen, and Pfizer, served on advisory boards for Gilead and has received institutional research funding from Stiftung Deutsche Krebshilfe and Merck KGaA.

References

1. Joseph P, Leong D, McKee M, Anand SS, Schwalm J-D, Teo K, *et al.* Reducing the Global Burden of Cardiovascular Disease, Part 1: The Epidemiology and Risk Factors. Circ Res 2017;121(6):677–94.
2. Bhaskaran A, De Silva K, Kumar S. Contemporary updates on ventricular arrhythmias: from mechanisms to management. Internal Medicine Journal 2023;53(6):892–906.
3. Grehn M, Mandija S, Miszczyk M, Krug D, Tomasik B, Stickney KE, *et al.* STereotactic Arrhythmia Radioablation (STAR): the Standardized Treatment and Outcome Platform for Stereotactic Therapy Of Re-entrant tachycardia by a Multidisciplinary consortium (STOPSTORM.eu) and review of current patterns of STAR practice in Europe. EP Europace 2023;25(4):1284–95.
4. Kornowski R. Patient-reported outcome measures in cardiovascular disease. European Heart Journal - Quality of Care and Clinical Outcomes 2023;9(2):119–27.
5. Miszczyk M, Hoeksema WF, Kuna K, Blamek S, Cuculich PS, Grehn M, *et al.* Stereotactic arrhythmia radioablation (STAR)—A systematic review and meta-analysis of prospective trials on behalf of the STOPSTORM.eu consortium. Heart Rhythm 2025;22(1):80–9.
6. Calvert M, Blazeby J, Altman DG, Revicki DA, Moher D, Brundage MD, *et al.* Reporting of patient-reported outcomes in randomized trials: the CONSORT PRO extension. JAMA 2013;309(8):814–22.
7. Anker SD, Agewall S, Borggrefe M, Calvert M, Jaime Caro J, Cowie MR, *et al.* The importance of patient-reported outcomes: a call for their comprehensive integration in cardiovascular clinical trials. European Heart Journal 2014;35(30):2001–9.
8. Savarese G, Lindenfeld J, Stolfo D, Adams K, Ahmad T, Desai NR, *et al.* Use of patient-reported outcomes in heart failure: from clinical trials to routine practice. Eur J Heart Fail

2023;25(2):139–51.
9. White J, Withers KL, Lencioni M, Carolan-Rees G, Wilkes AR, Wood KA, *et al.* Cardiff cardiac ablation patient-reported outcome measure (C-CAP): validation of a new questionnaire set for patients undergoing catheter ablation for cardiac arrhythmias in the UK. Qual Life Res 2016;25(6):1571–83.
10. Walfridsson U, Walfridsson H, Middeldorp ME, Sanders P, Årestedt K. Validation of the English version of the arrhythmia-specific questionnaire in tachycardia and arrhythmia (ASTA): a Rasch evaluation study. J Patient Rep Outcomes 2022;6(1):90.
11. Sandhu U, Nguyen AT, Dornblaser J, Gray A, Paladino K, Henrikson CA, *et al.* Patient-Reported Outcomes in a Multidisciplinary Electrophysiology-Psychology Ventricular Arrhythmia Clinic. J Am Heart Assoc 2022;11(15):e025301.
12. National Institutes of Health. NIH's Definition of a Clinical Trial; [cited 2025 Jun 30]. available at: https://grants.nih.gov/policy-and-compliance/policy-topics/clinical-trials/definition#background.
13. Peters MDJ, Godfrey C, McInerney P, Khalil H, Larsen P, Marnie C, *et al.* Best practice guidance and reporting items for the development of scoping review protocols. JBI Evidence Synthesis 2022;20(4):953.
14. Tricco AC, Lillie E, Zarin W, O'Brien KK, Colquhoun H, Levac D, *et al.* PRISMA Extension for Scoping Reviews (PRISMA-ScR): Checklist and Explanation. Ann Intern Med 2018;169(7):467–73.
15. Churruca K, Pomare C, Ellis LA, Long JC, Henderson SB, Murphy LED, *et al.* Patient-reported outcome measures (PROMs): A review of generic and condition-specific measures and a discussion of trends and issues. Health Expect 2021;24(4):1015–24.

Appendices

Appendix I: Search strategy

Appendix II: Data extraction instrument 1 (Published trials)

Appendix III: Data extraction instrument 2 (Unpublished registered trials)

Appendix I - Search Strategy

Database(s): **Ovid MEDLINE(R) and Epub Ahead of Print, In-Process, In-Data-Review & Other Non-Indexed Citations, Dail and Versions.** 1946 to September 03, 2025.
Search strategy executed on **04/09/2025**

#	Searches	Results
1	arrhythmias, cardiac/ or atrial fibrillation/ or atrial flutter/ or exp cardiac complexes, premature/ or bundle-branch block/ or interatrial block/ or parasystole/ or wolff-parkinson-white syndrome/ or exp tachycardia/ or ventricular fibrillation/ or ventricular flutter/	216824
2	heart conduction system/ or atrioventricular node/ or sinoatrial node/ or accessory atrioventricular bundle/	43163
3	pulmonary veins/re, su	7345
4	(((cardiac or heart or atri* or auric* or AV or A-V or ventric* or supraventr* or LA or LV or external or events or ectopic or reciproc* or junctional or sinus) adj9 (fibrillat* or flutter* or ar?hythm* or tachycard* or tachyar?h* or tachy-ar?h* or preexcitat* or pre-excitat*)) or (torsade adj2 point*) or AIVR or ((idioventr* or idio-ventr*) adj3 rhythm*)).tw,kf.	235853
5	ar?hythmogen*.tw,kf.	14551
6	((VT or VTs) adj2 (scar* or VF or AF or exit site* or epicard* or cardiomyo* or isch?em* or episod* or sustain* or burden or ablat* or radioablat* or radiosurg* or radio-surg*)).tw,kf.	5075
7	(((prematur* or pre-matur* or ectopic* or echo) adj3 (beat* or heartbeat* or ((cardiac or atrial or ventric*) adj2 (complex* or contraction*)))) or PVC-burden or PVCs or extra-systol* or extrasystol* or late potentials or ventricular LP*).tw,kf.	17083
8	(parasystol* or para-systol* or (electric* adj2 storm*) or ((slow* or intermediat*) adj2 (pathway* or junction* or conduction*))).tw,kf.	9497
9	(((AV or A-V or atrioventr* or atrio-ventr* or sinoatrial or sino-atrial or interatrial or inter-atrial or (card* adj3 conduct*)) adj6 (node or nodes or nodal or junction* or pathway* or conduct* or interrupt* or block* or reentr* or re-entr* or antegrad* or reciproc* or orthodromic* or rhythm*)) or ((epicard* or endocard*) adj3 exit) or (nodal adj2 reentr*)).tw,kf.	41687
10	(((accessory or alternativ*) adj3 (AV or A-V or atrio-ventr* or atrioventr* or connection or conduct*)) or (accessory adj3 pathway*) or AP-ablat* or ((parahis* or atriohis*) adj2 (bypass* or tract*)) or (bundle adj2 Kent) or (mahaim adj2 fib*) or ((heart or card*) adj2 conduction*)).tw,kf.	12002
11	(Wolff-Parkinson-White or WPW or ((ventric* or cardiac or syndrom*) adj6 (preexcitat* or excitat*)) or (bundle adj2 block*)).tw,kf.	20335
12	((((pulm* adj2 (vein or venous)) or PV or PVI or RSPV) adj4 (isolat* or tissue* or junction* or antrum or ostia or ostium or tissue or amplitud* or ablat* or radioablat* or radiosurg* or radio-surg* or radiat*)) or PVAI* or CPVI*).tw,kf.	9680
13	or/1-12 [cardiac arrhythmias]	365576
14	exp heavy ion radiotherapy/ or radiosurgery/ or exp radiotherapy, high-energy/ or exp radiotherapy, conformal/	65233

15	heavy ions/ or ((exp radiotherapy/ or radiation dosage/ or rt.fs.) and photons/)	5310
16	(exp radiotherapy, computer-assisted/ or radiotherapy, image-guided/) and (ablation techniques/ or exp arrhythmias, cardiac/rt or exp heart conduction system/re or photons/ or stereotaxic techniques/)	2157
17	(radioablat* or radiosurg* or (radio adj (ablat* or surg*)) or (radiother* adj5 ablat*)).tw,kf.	21235
18	((non-invasiv* or noninvasiv* or ablat*) adj2 cardiac adj1 (radiat* or irradiat*)).tw,kf.	4
19	((catheter* adj1 free) not (stuck* adj6 catheter*)).tw,kf.	437
20	((stereotact* and (radiother* or irradiat* or radiat* or ablat*)) or SBRT or SABR).tw,kf.	24379
21	(((multi-leaf or multileaf) adj2 collimator*) or MLC-track*).tw,kf.	2566
22	(((heavy or carbon or C12 or C-12 or 12C or 12-C) adj3 ion*) or THIR).tw,kf. not ((heavy adj3 metal*) or carbon dioxid*).mp.	9961
23	((photon* or proton* or ion* or particle* or carbon* or C12 or C-12 or 12C or 12-C) adj2 (beam* or irradiat*)).tw,kf.	28108
24	((proton* adj3 (therap* or radiother* or ablat*)) not proton-pump*).tw,kf.	9026
25	(Cyberknife or Cyber-knife or Cyberheart or Cyber-heart).tw,kf.	2218
26	or/14-25 [radioablation]	113249
27	((cancer* or carcinom* or adenocarcinom* or tumo?r* or neoplas* or malignan* or sarcom* or leiomyosarcom* or adenom* or metasta* or neoadjuv* or neo-adjuv* or chemother* or cytostat* or checkpoint inhib* or prostat* or breast* or mastect* or postmastect* or thorax or thoracic or mediastin* or lobectom* or mesotheliom* or NSCLC* or cholangiocarcinom* or lymphom* or leuk?em* or AML or melanom* or schwannom* or NF2 or neurofibrom* or paragangliom* or gliom* or glioblastom*) not (fibrillat* or flutter or arrhythm* or tachycard* or cardiac or sinus or atrioventr* or atrium or ventricul*)).ti,ot. [cancer (therapy) in title]	3605855
28	26 not 27 [radioablation (not cancer therapy as main topic)]	64390
29	13 and 28 [I cardiac arrhythmias & radiotherapy]	453
30	(RRPVI* or RR-PVI*).tw,kf. [II robotic radiosurgical pulmonary vein isolation]	1
31	((stereotact* or STAR) adj3 ar?hythmi* adj2 (radioablat* or radioablat*)).tw,kf.	101
32	30 or 31 [II robotic radiosurgical pulmonary vein isolation / STAR]	102
33	29 or 32 [I II cardiac arrhythmias & radiotherapy]	455
34	remove duplicates from 33 [I II cardiac arrhythmias & radiotherapy -deduplicated]	455

Database(s): **Embase via Embase.com**. Search strategy executed on: **04-09-2025.**

#	Searches	Results
#1	'arrhythmogenesis'/de OR 'heart arrhythmia'/de OR 'atrioventricular junction arrhythmia'/de OR 'experimental arrhythmia'/exp OR 'heart fibrillation'/de OR 'atrial fibrillation'/exp OR 'heart ventricle fibrillation'/exp OR 'tachycardia'/mj OR 'heart ventricle tachycardia'/de OR 'paroxysmal tachycardia'/exp OR 'polymorphic ventricular tachycardia'/de OR 'monomorphic ventricular tachycardia'/de OR 'reentry tachycardia'/exp OR 'sinus tachycardia'/de OR 'supraventricular tachycardia'/exp OR 'heart atrium arrhythmia'/de OR 'heart atrium flutter'/de OR 'heart supraventricular arrhythmia'/de OR 'supraventricular premature beat'/de OR 'heart ventricle arrhythmia'/de OR 'heart ventricle extrasystole'/de OR 'heart ventricle flutter'/de OR 'parasystole'/de OR 'reentry arrhythmia'/de OR 'heart muscle conduction disturbance'/de OR 'heart accessory conduction pathway'/de OR 'heart bundle branch block'/exp OR 'interatrial block'/de OR 'torsade des pointes'/de	596675
#2	('heart muscle conduction system'/exp OR 'heart conduction'/exp)	43944
#3	'pulmonary vein isolation'/de OR 'pulmonary veins'/de	30824
#4	(((cardiac OR heart OR atri* OR auric* OR AV OR A-V OR ventric* OR supraventr* OR LA OR LV OR external OR events OR ectopic OR reciproc* OR junctional OR sinus) NEXT/9 (fibrillat* OR flutter* OR ar?hythm* OR tachycard* OR tachyar?h* OR tachy-ar?h* OR preexcitat* OR pre-excitat*)) OR (torsade NEXT/2 point*) OR AIVR OR ((idioventr* OR idio-ventr*) NEXT/3 rhythm*)):ti,ab,kw	338315
#5	ar?hythmogen*:ti,ab,kw	21336
#6	((VT OR VTs) NEXT/2 (scar* OR VF OR AF OR exit-site* OR epicard* OR cardiomyo* OR isch?em* OR episod* OR sustain* OR burden OR ablat* OR radioablat* OR radiosurg* OR radio-surg*)):ti,ab,kw	8255
#7	(((prematur* or pre-matur* or ectopic* or echo) next/3 (beat* or heartbeat* or contraction*)) or pvc-burden or pvcs or extra-systol* or extrasystol* or late potentials or ventricular lp*):ti,ab,kw	4622
#8	(parasystol* OR para-systol* OR (electric* NEXT/2 storm*) OR ((slow* OR intermediat*) NEXT/2 (pathway* OR junction* OR conduction*))):ti,ab,kw	8644
#9	(('av':ti,ab,kw OR 'a-v':ti,ab,kw OR 'atrioventr*':ti,ab,kw OR 'atrio-ventr*':ti,ab,kw OR 'sinoatrial':ti,ab,kw OR 'sino-atrial':ti,ab,kw OR 'interatrial':ti,ab,kw OR 'inter-atrial':ti,ab,kw OR (('card*' NEAR/3 'conduct*'):ti,ab,kw)) AND ('node':ti,ab,kw OR 'nodes':ti,ab,kw OR 'nodal':ti,ab,kw OR 'junction*':ti,ab,kw OR 'pathway*':ti,ab,kw OR 'conduct*':ti,ab,kw OR 'interrupt*':ti,ab,kw OR 'block*':ti,ab,kw OR 'reentr*':ti,ab,kw OR 're-entr*':ti,ab,kw OR 'antegrad*':ti,ab,kw OR 'reciproc*':ti,ab,kw OR 'orthodromic*':ti,ab,kw OR 'rhythm*':ti,ab,kw) OR ((('epicard*' OR 'endocard*') NEAR/3 'exit'):ti,ab,kw) OR (('nodal' NEAR/2 'reentr*'):ti,ab,kw))	65319
#10	((('accessory' OR 'alternativ*') NEAR/3 ('AV' OR 'A-V' OR 'atrio-ventr*' OR 'atrioventr*' OR 'connection' OR 'conduct*')) OR ('accessory' NEAR/3 'pathway*') OR 'AP-ablat*' OR (('parahis*' OR 'atriohis*') NEAR/2 ('bypass*' OR 'tract*')) OR ('bundle' NEAR/2 'Kent') OR ('mahaim' NEAR/2 'fib*') OR (('heart' OR 'card*') NEAR/2 'conduction*')):ti,ab,kw	14590
#11	(Wolff-Parkinson-White or WPW or ((ventric* or cardiac or syndrom*) NEAR/6 (preexcitat* or excitat*)) or (bundle adj2 block*)):ti,ab,kw	7827

#12	(((('pulm*' NEXT/1 ('vein' OR 'venous')):ti,ab,kw) OR 'pv':ti,ab,kw OR 'pvi':ti,ab,kw OR 'rspv':ti,ab,kw) AND ('isolat*':ti,ab,kw OR 'tissue*':ti,ab,kw OR 'junction*':ti,ab,kw OR 'antrum':ti,ab,kw OR 'ostia':ti,ab,kw OR 'ostium':ti,ab,kw OR 'tissue':ti,ab,kw OR 'amplitud*':ti,ab,kw OR 'ablat*':ti,ab,kw OR 'radioablat*':ti,ab,kw OR 'radiosurg*':ti,ab,kw OR 'radio-surg*':ti,ab,kw OR 'radiat*':ti,ab,kw) OR 'pvai*':ti,ab,kw OR 'cpvi*':ti,ab,kw) AND [embase]/lim	16127
#13	#1 OR #2 OR #3 OR #4 OR #5 OR #6 OR #7 OR #8 OR #9 OR #10 OR #11 OR #12	699930
#14	'radiosurgery'/de OR 'gamma knife radiosurgery'/de OR 'stereotactic body radiation therapy'/de OR 'stereotactic radiosurgery'/de OR 'intensity modulated radiation therapy'/de OR 'ion therapy'/de OR 'photon therapy'/de OR 'proton therapy'/de	104860
#15	'radiosurgery equipment'/de OR 'gamma knife'/de OR 'cyberknife'/de OR 'multileaf collimator'/de	10847
#16	'megavoltage radiotherapy'/de OR 'heavy ions'/de	3144
#17	'ablation techniques'/de AND 'radiotherapy'/de	520
#18	('radioablat*':ti,ab,kw OR 'radiosurg*':ti,ab,kw OR (('radio' NEXT/0 ('ablat*' OR 'surg*')):ti,ab,kw) OR (('radiother*' NEXT/4 'ablat*'):ti,ab,kw))	26735
#19	((('non-invasiv*' OR 'noninvasiv*' OR 'ablat*') NEXT/2 'cardiac' NEXT/1 ('radiat*' OR 'irradiat*')):ti,ab,kw)	7
#20	('stereotact*':ti,ab,kw AND ('radiother*':ti,ab,kw OR 'irradiat*':ti,ab,kw OR 'radiat*':ti,ab,kw OR 'ablat*':ti,ab,kw) OR 'sbrt':ti,ab,kw OR 'sabr':ti,ab,kw)	42254
#21	((('multi-leaf' OR 'multileaf') NEXT/1 'collimator*') OR 'MLC-track*'):ti,ab,kw	3083
#22	((('heavy' OR 'carbon' OR 'C12' OR 'C-12' OR '12C' OR '12-C') NEXT/2 'ion*') OR 'THIR'):ti,ab,kw NOT (('heavy' NEXT/2 'metal*') OR 'carbon dioxid*')	7149
#23	((('photon*' OR 'proton*' OR 'ion*' OR 'particle*' OR 'carbon*' OR 'c12' OR 'c-12' OR '12c' OR '12-c') NEXT/1 ('beam*' OR 'irradiat*')):ti,ab,kw)	22589
#24	(('proton*' NEXT/2 ('therap*' OR 'radiother*' OR 'ablat*')) NOT 'proton-pump*'):ti,ab,kw	12292
#25	('Cyberknife' OR 'Cyber-knife' OR 'Cyberheart' OR 'Cyber-heart'):ti,ab,kw	3833
#26	#14 OR #15 OR #16 OR #17 OR #18 OR #19 OR #20 OR #21 OR #22 OR #23 OR #24 OR #25	142898
#27	(('cancer*' OR 'carcinom*' OR 'adenocarcinom*' OR 'tumor' OR 'neoplas*' OR 'malignan*' OR 'sarcom*' OR 'leiomyosarcom*' OR 'adenom*' OR 'metasta*' OR 'neoadjuv*' OR 'neo-adjuv*' OR 'chemother*' OR 'cytostat*' OR 'checkpoint inhib*' OR 'prostat*' OR 'breast*' OR 'mastect*' OR 'postmastect*' OR 'thorax' OR 'thoracic' OR 'mediastin*' OR 'lobectom*' OR 'mesotheliom*' OR 'NSCLC*' OR 'cholangiocarcinom*' OR 'lymphom*' OR 'leukem' OR 'AML' OR 'melanom*' OR 'schwannom*' OR 'NF2' OR 'neurofibrom*' OR 'paragangliom*' OR 'gliom*' OR 'glioblastom*') NOT ('fibrillat*' OR 'flutter' OR 'arrhythm*' OR 'tachycard*' OR 'cardiac' OR 'sinus' OR 'atrioventr*' OR 'atrium' OR 'ventricul*')):ti	4488210
#28	#26 NOT #27	69078
#29	#13 AND #28	1216

Database(s): **Web of Science (Core Collection).** Search strategy executed on **04-09-2025**

#	Searches	Results
#1	TS=(((cardiac OR heart OR atri* OR auric* OR AV OR A-V OR ventric* OR supraventr* OR LA OR LV OR external OR events OR ectopic OR reciproc* OR junctional OR sinus) NEAR/9 (fibrillat* OR flutter* OR arrhythm* OR tachycard* OR tachyarrh* OR tachy-arrh* OR preexcitat* OR pre-excitat*)) OR (torsade NEAR/2 point*) OR AIVR OR ((idioventr* OR idio-ventr*) NEAR/3 rhythm*) OR arrhythmogen* OR ((VT OR VTs) NEAR/2 (scar* OR VF OR AF OR exit-site* OR epicard* OR cardiomyo* OR isch*em* OR episod* OR sustain* OR burden OR ablat* OR radioablat* OR radiosurg* OR radio-surg*)) or ((prematur* or pre-matur* or ectopic* or echo) NEAR/3 (beat* or heartbeat*)) or ((cardiac or atrial or ventric*) NEAR/2 (complex* or contraction*)) or PVC-burden or PVCs or extra-systol* or extrasystol* or late-potentials or ventricular-LP* or parasystol* or para-systol* or (electric* NEAR/2 storm*) or ((slow* or intermediat*) NEAR/2 (pathway* or junction* or conduction*)) or ((AV or A-V or atrioventr* or atrio-ventr* or sinoatrial or sino-atrial or interatrial or inter-atrial or (card* NEAR/3 conduct*)) NEAR/6 (node or nodes or nodal or junction* or pathway* or conduct* or interrupt* or block* or reentr* or re-entr* or antegrad* or reciproc* or orthodromic* or rhythm*)) or ((epicard* or endocard*) NEAR/3 exit) or (nodal NEAR/2 reentr*) or ((accessory or alternativ*) NEAR/3 (AV or A-V or atrio-ventr* or atrioventr* or connection or conduct*)) or (accessory NEAR/3 pathway*) or AP-ablat* or ((parahis* or atriohis*) NEAR/2 (bypass* or tract*)) or (bundle NEAR/2 Kent) or (mahaim NEAR/2 fib*) or ((heart or card*) NEAR/2 conduction*) or Wolff-Parkinson-White or WPW or ((ventric* or cardiac or syndrom*) NEAR/6 (preexcitat* or excitat*)) or (bundle NEAR/2 block*) or (((pulm* NEAR/2 (vein or venous)) or PV or PVI or RSPV) NEAR/4 (isolat* or tissue* or junction* or antrum or ostia or ostium or tissue or amplitud* or ablat* or radioablat* or radiosurg* or radio-surg* or radiat*)) or PVAI* or CPVI*)	373,891
#2	TS=(radioablat* OR radiosurg* OR radio-ablat* OR radio-surg* OR (radiother* NEAR/5 ablat*) OR ((non-invasiv* OR noninvasiv* OR ablat*) NEAR/2 cardiac NEAR/1 (radiat* OR irradiat*)) OR ((catheter* NEAR/1 free) NOT (stuck* NEAR/6 catheter*)) OR (stereotact* AND (radiother* OR irradiat* OR radiat* OR ablat*)) OR SBRT OR SABR OR ((multi-leaf OR multileaf) NEAR/2 collimator*) OR MLC-track* OR ((((heavy OR carbon OR C12 OR C-12 OR 12C OR 12-C) NEAR/3 ion*) OR THIR) NOT ((heavy NEAR/3 metal*) OR carbon-dioxid*)) OR ((photon* OR proton* OR ion* OR particle* OR carbon* OR C12 OR C-12 OR 12C OR 12-C) NEAR/2 (beam* OR irradiat*)) OR ((proton* NEAR/3 (therap* OR radiother* OR ablat*)) NOT proton-pump*) OR Cyberknife OR Cyber-knife OR Cyberheart OR Cyber-heart)	298,469
#3	#1 AND #2	648
#4	TI=((cancer* OR carcinom* OR adenocarcinom* OR tumor* OR tumour* OR neoplas* OR malignan* OR sarcom* OR leiomyosarcom* OR adenom* OR metasta* OR neoadjuv* OR (neo-adjuv*) OR chemother* OR cytostat* OR (checkpoint-inhib*) OR prostat* OR breast* OR mastect* OR postmastect* OR thorax OR thoracic OR mediastin* OR lobectom* OR mesotheliom* OR NSCLC* OR cholangiocarcinom* OR lymphom* OR leukem* OR leukaem* OR AML OR melanom* OR schwannom* OR NF2 OR neurofibrom* OR paragangliom* OR gliom* OR glioblastom*) NOT (fibrillat* OR flutter OR arrhythm* OR tachycard* OR cardiac OR sinus OR atrioventr* OR atrium OR ventricul*))	4,544,808
#5	#3 NOT #4	611

Database(s): **Cochrane Central Register of Controlled Trials.** Search strategy executed on: **04-09-2025**

#	Searches	Results
1	(((cardiac OR heart OR atri* OR auric* OR AV OR (A adj V) OR ventric* OR supraventr* OR LA OR LV OR external OR events OR ectopic OR reciproc* OR junctional OR sinus) adj9 (fibrillat* OR flutter* OR arrhythm* OR tachycard* OR tachyarrh* OR (tachy adj arrh*) OR preexcitat* OR (pre adj excitat*))) OR (torsade adj2 point*) OR AIVR OR ((idioventr* OR (idio adj ventr*)) adj3 rhythm*)).ti,ab,kw	27851
2	arrhythmogen*.ti,ab,kw	481
3	((VT OR VTs) adj2 (scar* OR VF OR AF OR (exit adj site*) OR epicard* OR cardiomyo* OR ischem* OR ischaem* OR episod* OR sustain* OR burden OR ablat* OR radioablat* OR radiosurg* OR (radio adj surg*))).ti,ab,kw	611
4	(((prematur* OR (pre adj matur*) OR ectopic* OR echo) adj3 (beat* OR heartbeat* OR ((cardiac OR atrial OR ventric*) adj2 (complex* OR contraction*)))) OR (PVC adj burden) OR PVCs OR (extra adj systol*) OR extrasystol* OR (late adj potentials) OR (ventricular adj LP*)).ti,ab,kw	1850
5	(parasystol* OR (para adj systol*) OR (electric* adj2 storm*) OR ((slow* OR intermediat*) adj2 (pathway* OR junction* OR conduction*))).ti,ab,kw	205
6	(((AV OR (A adj V) OR atrioventr* OR (atrio adj ventr*) OR sinoatrial OR (sino adj atrial) OR interatrial OR (inter adj atrial) OR (card* adj3 conduct*)) adj6 (node OR nodes OR nodal OR junction* OR pathway* OR conduct* OR interrupt* OR block* OR reentr* OR (re adj entr*) OR antegrad* OR reciproc* OR orthodromic* OR rhythm*)) OR ((epicard* OR endocard*) adj3 exit) OR (nodal adj2 reentr*)).ti,ab,kw	2847
7	(((accessory OR alternativ*) adj3 (AV OR (A adj V) OR (atrio adj ventr*) OR atrioventr* OR connection OR conduct*)) OR (accessory adj3 pathway*) OR (AP adj ablat*) OR ((parahis* OR atriohis*) adj2 (bypass* OR tract*)) OR (bundle adj2 Kent) OR ((heart OR card*) adj2 conduction*)).ti,ab,kw	698
8	(Wolff adj Parkinson adj White OR WPW OR ((ventric* OR cardiac OR syndrom*) adj6 (preexcitat* OR excitat*)) OR (bundle adj2 block*)).ti,ab,kw	1012
9	((((pulm* adj2 (vein OR venous)) OR PV OR PVI OR RSPV) adj4 (isolat* OR tissue* OR junction* OR antrum OR ostia OR ostium OR tissue OR amplitud* OR ablat* OR radioablat* OR radiosurg* OR (radio adj surg*) OR radiat*)) OR PVAI* OR CPVI*).ti,ab,kw	2133
10	1 or 2 or 3 or 4 or 5 or 6 or 7 or 8 or 9	31235
11	(radioablat* OR radiosurg* OR (radio adj (ablat* OR surg*)) OR (radiother* adj5 ablat*)).ti,ab,kw	1428
12	(((non adj invasiv*) OR noninvasiv* OR ablat*) adj2 cardiac adj1 (radiat* OR irradiat*)).ti,ab,kw	0
13	((catheter* adj1 free) NOT (stuck* adj6 catheter*)).ti,ab,kw	55
14	((stereotact* AND (radiother* OR irradiat* OR radiat* OR ablat*)) OR SBRT OR SABR).ti,ab,kw	2558
15	(((multi adj leaf OR multileaf) adj2 collimator*) OR (MLC adj track*)).ti,ab,kw	54
16	(((heavy OR carbon OR C12 OR (C adj 12) OR 12C OR (12 adj C)) adj3 ion*) OR THIR).ti,ab,kw NOT ((heavy adj3 metal*) OR (carbon adj dioxid*)).ti,ab,kw	191
17	((photon* OR proton* OR ion* OR particle* OR carbon* OR C12 OR (C adj 12) OR 12C OR (12 adj C)) adj2 (beam* OR irradiat*)).ti,ab,kw	507
18	((proton* adj3 (therap* OR radiother* OR ablat*)) NOT (proton adj pump*)).ti,ab,kw	537
19	(Cyberknife or (Cyber NEXT knife) or Cyberheart or (Cyber NEXT heart)):ti,ab,kw	88

20	11 or 12 or 13 or 14 or 15 or 16 or 17 or 18 or 19	4041
21	(cancer* OR carcinom* OR adenocarcinom* OR tumor* OR tumour* OR neoplas* OR malignan* OR sarcom* OR leiomyosarcom* OR adenom* OR metasta* OR neoadjuv* OR (neo adj adjuv*) OR chemother* OR cytostat* OR (checkpoint adj inhib*) OR prostat* OR breast* OR mastect* OR postmastect* OR thorax OR thoracic OR mediastin* OR lobectom* OR mesotheliom* OR NSCLC* OR cholangiocarcinom* OR lymphom* OR leukem* OR leukaem* OR AML OR melanom* OR schwannom* OR NF2 OR neurofibrom* OR paragangliom* OR gliom* OR glioblastom*) NOT (fibrillat* OR flutter OR arrhythm* OR tachycard* OR cardiac OR sinus OR atrioventr* OR atrium OR ventricul*).ti	369514
22	10 and 20	43
23	22 not 21	33

Database: **"ClinicalTrials.gov".** Search strategy executed on: **04-09-2025**
Condition: Ventricular Arrythmia OR Ventricular Tachycardia
Other terms: n/a
Intervention/treatment: radiotherapy OR radiosurgery OR stereotactic body radiotherapy OR SBRT OR radioablation
